# Steroid responsive encephalopathy associated with autoimmune thyroiditis (SREAT) presenting as subacute psychosis: Case report and literature review

**DOI:** 10.1002/ccr3.8848

**Published:** 2024-05-10

**Authors:** Elabbass A. Abdelmahmuod, Noor Jasim, Buthaina Ibrahim, Marwa Mokhtar

**Affiliations:** ^1^ Internal Medicine Department Hamad Medical Corporation Doha Qatar; ^2^ Endocrine Department Hamad Medical Corporation Doha Qatar

**Keywords:** Hashimoto, hypothyroidism, psychosis, thyroiditis

## Abstract

Clinicians should consider autoimmune thyroiditis in patients presenting with neuropsychiatric symptoms and promptly initiate appropriate investigations and treatment, such as corticosteroids, to improve clinical outcomes.

## 1 INTRODUCTION

Steroid response encephalopathy associated with autoimmune thyroiditis (SREAT) has the potential to manifest abruptly with numerous recurring focal neurological episodes or gradually with a widespread, diffuse configuration distinguished by cognitive impairment.^1^ It is not adequately acknowledged by medical practitioners, and the objective of this account is to contribute to the existing body of knowledge and emphasize the possibility of a favorable clinical prognosis when employing appropriate therapeutic measures.^2^

The prevalence of Hashimoto's encephalopathy is not adequately established and continues to be overlooked. Primarily, steroids are employed as the first‐line treatment, however, the initial dosage, duration of treatment, and factors related to treatment failure are yet to be determined. Despite reports of steroid dependency and a heightened relapse rate, the role of steroid‐sparing treatments remains undetermined.^3^

Steroid‐responsive encephalitis associated with autoimmune thyroiditis (SREAT) is a condition that necessitates the prompt initiation of steroids, as they serve as the primary treatment. In the majority of instances, treatment results in significant amelioration of symptoms. However, the efficacy of alternative immunosuppressive medications, particularly in cases where there is inadequate response to steroids, remains uncertain.^4^

## 2 CASE HISTORY

A 21‐year‐old gentleman presented with a 1‐month history of abnormal behavior, including auditory hallucinations, persecutory delusions, and paranoia. He also reported chronic constipation, insomnia, fatigue, cold intolerance, low mood, increased isolation, and poor concentration. There was no history of substance abuse or family thyroid disease. Physical examination revealed a mildly enlarged thyroid without nodules and normal neurological findings. Mental status examination showed low mood, slow speech, paranoid delusions, and fleeting self‐harm thoughts without a plan for self‐harm.

### 2.1 Investigations

Laboratory tests indicated significantly elevated TSH (>100 IU/mL) {0.3–4.20 IU/mL}‐and low FT4 levels (0.8 pmol/L){11–23.4 pmol/L}, along with high anti‐thyroid peroxidase antibodies (TPO) level was 400 IU/mL. Imaging ruled out space‐occupying lesions or infections. CSF analysis was normal, with increased IgG levels.

### 2.2 Diagnosis

A diagnosis of Hashimoto's encephalopathy (HE) or steroid‐responsive encephalopathy associated with autoimmune thyroiditis (SREAT) was made, given the exclusion of encephalitis and the patient's remarkable improvement upon treatment.

### 2.3 Treatment

Initial treatment included one dose of risperidone 2 mg, as schizophrenia was initially suspected. Subsequently, the patient received daily levothyroxine (100 mcg) after thyroid function tests. IV methylprednisolone 20 mg was administered for 5 days, resulting in significant symptom improvement. The improvement occurred within just 2 days following the confirmation of diagnosis and starting the management.

### 2.4 Follow‐up

The patient was discharged with plans for outpatient follow‐up and repeat thyroid function tests in 4–6 weeks. By the time of discharge, the patient demonstrated notable progress in his recovery journey. He expressed readiness to resume his study commitments and exhibited improved social interactions with both family members and friends.

## 3 DISCUSSION

Hashimoto's encephalopathy continues to be a topic of debate ever since it was first described in 1966. The precise cause of this condition has yet to be fully understood, although it is believed to be autoimmune in nature.^5^ There is currently no consensus on the specific autoimmune response involved. Several mechanisms of disease have been proposed, including primary demyelination, vasculitis, immune complex deposition, and direct antibody‐mediated neuronal injury. Symptoms of Hashimoto's encephalopathy include confusion, with or without myoclonus, seizures, hyperreflexia, and psychosis. The condition may present as a gradual onset of cognitive impairment or as recurrent episodes of focal neurological deficit accompanied by confusion.^6^

Reports have come across patients presenting with seizures, myoclonus, tremors, and cognitive impairment. Other articles reported atypical presentations such as depression, catatonia, or hallucinations. The variety of symptoms can be an obstacle in distinguishing HE/SREAT from neurologic/psychiatric conditions.^7^ To date, HE/SREAT is identified by the presence of encephalopathy with the exclusion of systemic disease and infectious causes. Concurrently, patients should have positive anti‐thyroid peroxidase antibodies (TPO) and show marked response to steroid therapy.^8^

Our case offers additional evidence regarding the role of anti‐TPO in the classification of HE/SREAT. Currently, the detection of elevated anti‐thyroid antibodies is the primary diagnostic finding for HE/SREAT.^9^ The majority of reported cases have shown the presence of anti‐TPO, anti‐thyroglobulin antibodies, and thyroid microsomal antibodies. Interestingly, it has been observed that the level of antibodies does not correlate with the severity of the disease. It is important to note that while our case exhibited severe hypothyroidism, abnormal thyroid function does not contribute to the diagnosis of HE/SREAT. In fact, patients have been reported as HE/SREAT with normal thyroid function tests or have developed abnormalities in thyroid function at a later stage.^10^

Comparatively, as demonstrated in our case, neuroimaging such as MRI was normal, which was concurrently reported in most cases. CSF analyses were mainly revealed to have high IgG and titers of anti‐thyroid antibodies, although the impact of this finding in relation to pathogenesis is not clear.^11^ In other reports, EEG studies were abnormal, showing generalized slow‐wave abnormalities. However, the EEG findings did not correlate with the results of MRI/CT and thus are not part of the diagnostic criteria of HE/SREAT. Moreover, once patients clinically improved, repeat EEG findings were reported to improve and return to normal.^12^

Undoubtedly, the majority of cases have demonstrated the efficacy of steroid therapy, which is why it is regarded as the primary approach in managing patients suspected of having HE/SREAT. However, there is yet to be a consensus on the optimal dosage and duration of treatment. According to most reports, patients have shown clinical improvement either with the administration of 1 g of intravenous methylprednisolone for 5–7 days or with oral prednisolone ranging from 30 to 100 mg, followed by a gradual tapering over a period of weeks to months. In cases where there is a poor or adverse response to steroid therapy, immune modulators like azathioprine, methotrexate, and mycophenolate may be added, or alternatively, intravenous immunoglobulin (IVIG), and plasmapheresis could be substituted.

Currently, the greatest discrepancy lies in the origin and pathophysiology of HE/SREAT. The existing body of literature agrees that the underlying cause of the disease is rooted in an autoimmune response. It is hypothesized that the presence of immune complex deposition in both the thyroid gland and brain, along with a shared antigen, is responsible for the clinical manifestations of HE/SREAT.^3^ However, there is currently no established link between the central nervous system (CNS) and the thyroid gland. Discussions in the field speculate that there may be an unidentified marker present in the brain and thyroid, which could explain the pathogenic nature of this syndrome. Currently, the only marker discovered in patients with HE/SREAT is alpha‐enolase, which is also found in various autoimmune vasculitis diseases. This marker may provide insights into the pathogenesis of the disease, but further study is needed to fully understand its implications. Nevertheless, this evidence supports the prevailing theories regarding autoimmune vasculitis as the primary mechanism behind the development of the disease.^12^

Another theory suspects that HE/SREAT develops on the basis of dysregulated hormone production at the level of the hypothalamus. This idea is based on dysregulation of TSH, which in a state of hypothyroidism can lead to hyperprolactinemia, leading to a risk of autoimmunity.^13^ However, this speculation alone would not explain the basis of the disease, as we previously mentioned that not all patients have abnormal thyroid function when presenting with HE/SREAT. On the other hand, pathologic analysis of brain tissue is also limited and gives no evidence of specific characteristics, as patients improve after steroid therapy by the time a sample is taken. The samples mainly showed lymphocytes supporting the presence of vasculitis disease. Perhaps the pathogenesis of HE/SREAT involves an amalgamation of multiple systemic factors alongside the established element of autoimmunity.^14^

## 4 CONCLUSION

There is still a significant amount of knowledge that needs to be acquired with regard to the pathogenesis of Hashimoto's encephalopathy. It is crucial to include this condition in the process of differential diagnosis for any metabolic encephalopathy, as timely intervention can result in a favorable outcome and complete restoration of health.

## AUTHOR CONTRIBUTIONS

**Elabbass A. Abdelmahmuod:** Investigation; writing – original draft; writing – review and editing. **Noor Jasim:** Writing – original draft; writing – review and editing. **Buthaina Ibrahim:** Writing – original draft; writing – review and editing. **Marwa Mokhtar:** Supervision; writing – original draft; writing – review and editing.

## FUNDING INFORMATION

This work was funded by Qatar National library.

## CONFLICT OF INTEREST STATEMENT

The authors have no conflicts of interest.

## ETHICS STATEMENT

This case was approved by the Hamad Medical Corporation's Medical Research Center, and the patient consented to the publication of his case.

## CONSENT

Written informed consent was obtained from the patient to publish this report in accordance with the journal's patient consent policy.

## Data Availability

The data that support the findings of this study are available from the corresponding author upon reasonable request.
